# The role of RNA epigenetic modification-related genes in the immune response of cattle to mastitis induced by *Staphylococcus aureus*

**DOI:** 10.5713/ab.23.0323

**Published:** 2024-01-20

**Authors:** Yue Xing, Yongjie Tang, Quanzhen Chen, Siqian Chen, Wenlong Li, Siyuan Mi, Ying Yu

**Affiliations:** 1 Key Laboratory of Animal Genetics, Breeding and Reproduction, Ministry of Agriculture & National Engineering Laboratory for Animal Breeding, College of Animal Science and Technology, China Agricultural University, Beijing 100193, China

**Keywords:** Mastitis, RNA Modification Gene, RNA-seq, *Staphylococcus aureus*

## Abstract

**Objective:**

RNA epigenetic modifications play an important role in regulating immune response of mammals. Bovine mastitis induced by *Staphylococcus aureus* (*S. aureus*) is a threat to the health of dairy cattle. There are numerous RNA modifications, and how these modification-associated enzymes systematically coordinate their immunomodulatory effects during bovine mastitis is not well reported. Therefore, the role of common RNA modification-related genes (RMRGs) in bovine *S. aureus* mastitis was investigated in this study.

**Methods:**

In total, 80 RMRGs were selected for this study. Four public RNA-seq data sets about bovine *S. aureus* mastitis were collected and one additional RNA-seq data set was generated by this study. Firstly, quantitative trait locus (QTL) database, transcriptome-wide association studies (TWAS) database and differential expression analyses were employed to characterize the potential functions of selected enzyme genes in bovine *S. aureus* mastitis. Correlation analysis and weighted gene co-expression network analysis (WGCNA) were used to further investigate the relationships of RMRGs from different types at the mRNA expression level. Interference experiments targeting the m^6^A demethylase *FTO* and utilizing public MeRIP-seq dataset from bovine Mac-T cells were used to investigate the potential interaction mechanisms among various RNA modifications.

**Results:**

Bovine QTL and TWAS database in cattle revealed associations between RMRGs and immune-related complex traits. *S. aureus* challenged and control groups were effectively distinguished by principal component analysis based on the expression of selected RMRGs. WGCNA and correlation analysis identified modules grouping different RMRGs, with highly correlated mRNA expression. The m^6^A modification gene *FTO* showed significant effects on the expression of m^6^A and other RMRGs (such as *NSUN2*, *CPSF2*, and *METTLE*), indicating complex co-expression relationships among different RNA modifications in the regulation of bovine *S. aureus* mastitis.

**Conclusion:**

RNA epigenetic modification genes play important immunoregulatory roles in bovine *S. aureus* mastitis, and there are extensive interactions of mRNA expression among different RMRGs. It is necessary to investigate the interactions between RNA modification genes regulating complex traits in the future.

## INTRODUCTION

*Staphylococcus aureus* (*S. aureus*) is a gram-positive opportunistic pathogen [1], and a common cause of mastitis in ruminants, which is a key concern of the dairy industry due to its wide range of species, complex mechanisms of action, and difficulty of cure [2]. Bovine mammary gland tissue and mammary alveolar cells, particularly bovine mammary epithelial cells (Mac-T cells), are popular experimental models in *S. aureus* mastitis studies [3] and play a critical role in bovine immunological defense [4]. The Mac-T cell line is an immortalized version of bovine mammary epithelial cells, established by introducing the SV-40 large T-antigen into primary bovine mammary alveolar cells [5,6]. The Mac-T cell line retains the typical ‘cobblestone’ structure and specific secretory functions of mammary epithelial cells (MECs), providing a more manageable model of MECs [4,7]. Coupling with its ability to clarify the immunological functions of MECs, the Mac-T cell line is widely used in *S. aureus* mastitis studies.

RNA epigenetic modification is widespread in mammals and contributes to many cellular biological processes such as immunity, lipid metabolism, biological rhythms, and reproductive development [8,9]. Most studies only investigated the regulatory roles of individual RNA-modifying enzymes or single modifications in complex traits. However, it has been shown that RNA modifications do not work independently, and there are extensive interactions among them [2,10]. RNA epigenetic modifications are mostly reversible, and coordinated with writers (methylation transferases), erasers (demethylases), and readers (proteins that bind specifically to methylation sites) [11].

RNA epigenetic modifications mainly include N6-methyladenosine (m^6^A), N6, 2’-O-dimethyl adenosine (m^6^Am), N1-methyladenosine (m^1^A), 5-methylcytosine (m^5^C), 5-hydroxymethylcytosine (hm^5^C), N4-acetyl cytidine (ac^4^C), alternative polyadenylation (APA), pseudouridine (Ψ), and N7-methylguanosine (m^7^G) [12–21]. It has been reported that the RNA epigenetic modifications extensively involved in the regulation of immune processes. For example, the m^6^A modification crucially regulates immune cell proliferation, differentiation, and function [12]. The m^1^A modification modulates T-cell immune responses and suppresses inflammation [18]. The hm^5^C modification potentially impacts immune cell signaling and inflammation, influencing the pathogenesis of immune-related diseases [22]. To understand the effects of RNA modification-related genes (RMRGs) on bovine *S. aureus* mastitis, we compiled a catalogue of genes linked to RNA modifications and inflammation as well as disease (Supplementary Table S1). Then, 80 RMRGs [12–21] were selected for this study by comparing with the bovine gene database.

In this study, we investigated the roles of RMRGs in bovine mastitis caused by *S. aureus* using principal component analysis (PCA), weighted gene co-expression network analysis (WGCNA), and correlation analyses. Furthermore, we validated the potential interaction mechanisms of RNA interference. This study on RNA epigenetic changes in the pathogenesis of *S. aureus* mastitis will be beneficial for reducing the incidence of bovine *S. aureus* mastitis and promoting the long-term development of the dairy industry.

## MATERIALS AND METHODS

### Design

**Figure 1:**
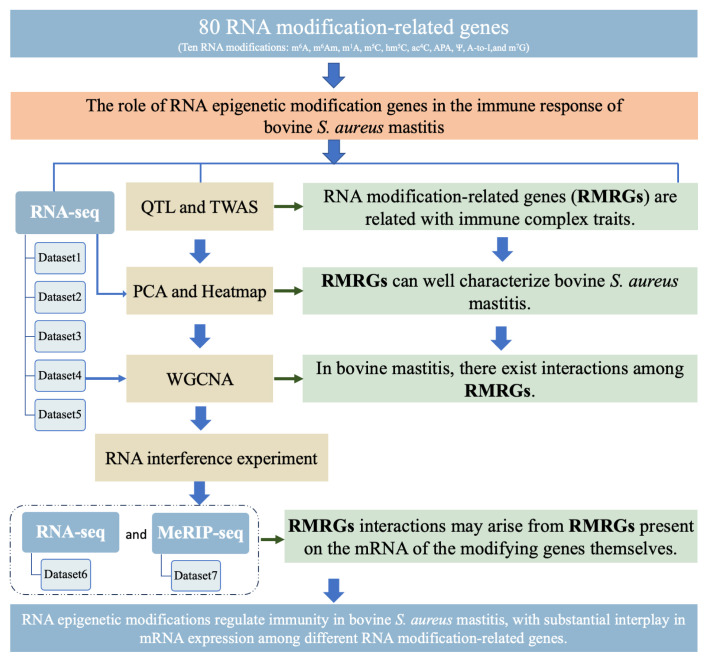
Technical route of this study.

To elucidate the roles of RNA epigenetic modification genes in bovine *S. aureus* mastitis, four types of analyses were performed in this study (Figure 1), including mapping analysis of 80 RMRGs using the animal quantitative trait locus (QTL) and cattle Genotype-Tissue Expression atlas (cGTEx) transcriptome-wide association studies (TWAS) databases, PCA and expression analysis based on five RNA sequencing (RNA-seq) datasets, WGCNA analysis based on dataset four. Additionally, validation of expression regulation was performed by interference experiments (dataset six) and methylated RNA immunoprecipitation sequencing (MeRIP-seq) data (dataset seven). The findings provide evidence for potential interactions among RNA modification genes, highlighting their involvement in the pathogenesis of bovine *S. aureus* mastitis.

### Data

To obtain public dataset on the transcriptional landscape of bovine *S. aureus* mastitis and m^6^A site information in cattle, the searches were performed on the NCBI GEO database platform using the terms “Mac-T cells”, “cattle”, “m^6^A”, and “*Staphylococcus aureus*”. In this study, a total of seven datasets were used in analyses, including five public datasets and two datasets generated by the current study. Of which, four raw RNA-seq datasets and one raw MeRIP-seq dataset were downloaded from public database. The underlined two datasets (five and six) were generated by the current study (Table 1).

Dataset one to five are related to *S. aureus* infection challenge compared to control. Based on various datasets, the samples from the control group (C) and *S. aureus* strain challenge group (S) were used in this study. Dataset one included the saline-treated bovine udder quarter (samples C-1 and C-2), and the high-concentration *S. aureus*-challenge udder quarter (samples S-1 and S-2). Dataset two comprised three groups, including the control group (samples C-1, C-2, and C-3); the Methicillin-Resistant *S. aureus* (MRSA) group (samples S1–1, S1–2, and S1–3); and the Methicillin-Sensitive *S. aureus* (MSSA) group (samples S2–1, S2–2, and S2–3) of the *S. aureus* challenge treatment. Dataset four included the control group (samples C-1, C-2 and C-3) and the MSSA-challenged group (samples S-1, S-2 and S-3). Dataset four generated by our previous study [3], included the control group (samples C-1 to C-6), the *S. aureus* strains group 1 (samples S1–1 to S1–6), *S. aureus* strains group 2 (samples S2–1 to S2–6), and *S. aureus* strains group 3 (samples S3–1 to S3–6). Dataset five generated by the current study, included control group (samples C-1 to C-4) and Newman strain of *S. aureus* challenged group (sample S-1 to S-4).

Dataset six is related to post-*siFTO* at the cellular level. In addition, the dataset seven is a MeRIP-seq dataset with three samples.

In addition, QTLs information covering all traits for cattle (ARS UCD1.2), pig (SS11.1), and sheep (OAR rambo1) were downloaded from the Animal QTL database ( www.animalgenome.org/cgi-bin/QTLdb/index ). Trait information predicted by cattle TWAS from the cGTEx database (cgtex.roslin. ed.ac.uk) was used in this study. The mapping analysis was used to integrate the RMRGs with the trait information from the QTLs database and the cattle TWAS data.

### RNA-seq data processing

The quality of the raw reads from the RNA-seq data was assessed using FastQC v0.11.9 ( https://www.bioinformatics.babraham.ac.uk/projects/fastqc/). Subsequently, the NGS QC Toolkit v2.3.3 [23] was used to remove adaptors and filter out poor-quality reads. The clean reads were aligned to the Bos taurus reference genome (version: ARS-UCD1.2.109) using the Hisat2 v2.2.1 [24]. The aligned SAM files were then converted into BAM files using SAMtools v1.9 ( https://github.com/samtools/samtools/releases/), followed by quantitative analysis with Featurecounts.

### Differentially expressed genes analysis

The DESeq2 tool ( https://bioconductor.org/packages/release/bioc/html/DESeq2.html ) in R software was used for data normalization across different samples, and the ComBat package [25] was used to correct batch effects. The DESeq2 tool was employed to obtain differentially expressed genes (DEGs) among different groups, which was determined by implementing the Wald test. Additionally, p-values were adjusted using the Benjamini-Hochberg method. A significance level of p<0.05 was considered to be statistically significant [26].

### m6A peak identification from MeRIP-seq data

Conversion of sra files to fasta format was executed with the SRA-Toolkit v.2.9.6 6 ( https://www.ncbi.nlm.nih.gov/books/NBK158900/). Contaminated reads with adapters, those of low quality, and ambiguous reads were removed using the NGS QC Toolkit v2.3.3 [23]. Then, the reads contained rRNA were removed using Bowtie2 v2.4.1 ( https://bowtie-bio.sourceforge.net/bowtie2/index.shtml ). Finally, the clean reads were mapped to the bovine reference genome using Hisat2 v2.2.1 [24]. The SAM files were then converted into BAM files using SAMtools v1.9, with m6A peaks called using the exomepeak2 package in R.

### Weighted gene co-expression network analysis

The gene co-expression network was constructed using the WGCNA package in R to detect the correlations among RMRGs. Considering the relatively large data size, dataset four was selected to perform WGCNA.

The top 10,000 highly variable genes were initially selected for WGCNA. Based on a soft threshold power for co-expression similarity, the adjacency was calculated to convert the adjacency matrix into a topology overlap matrix (TOM), and the corresponding difference (1-TOM) was calculated by using the TOM to represent the distance of the relationships between genes. The clustering of genes was then carried out using the 1-TOM. Thirdly, gene modules made up of at least 60 genes are created using dynamic cut-tree function detection and hierarchical clustering to group genes with comparable expression patterns. Finally, the threshold merge CutHeight parameter for merging modules was set to 0.25, and the key module was determined based on the DEGs between the *S. aureus* challenged group and the control group.

### Expression profile and correlation analysis

By employing DESeq2 and Combat [25] in R, read counts were normalization correction standardized. PCA results were shown with the ggplot2 ( https://ggplot2.tidyverse.org/) program, which was colored by distinct groupings. PCA was used to analyze specific changes in the expression of RMRGs in individual level of cattle and Mac-T cells after treatment.

Correlation heatmaps of mRNA expressions among various RNA epigenetic modifying enzymes were generated using Corrplot program in R. The Pearson correlations of RMRGs expressions among the control group in dataset four were calculated using the cor.test package in R. The hierarchical clustering (hclust) order technique was used to rank the correlation coefficients for Corplot plotting.

### Functional and pathway enrichment analysis

Kyoto encyclopedia of genes and genomes (KEGG) and gene ontology (GO) enrichment analysis were conducted on the gene modules of WGCNA and the DEGs following *FTO* interference by using KOBAS 3.0 ( https://bioinfo.org/kobas/genelist/). The R package ImageGP and ggplot2 were used to visualize 20 GO terms with a significance level of p<0.05.

### Cell culture, *S. aureus* challenged of Mac-T Cells, siRNA transfection

All the cells were grown at 37°C and 5% CO_2_. Mac-T cells were grown in the complete medium (Dulbecco’s modified eagle medium [DMEM]) supplemented with 10% fetal bovine serum and a penicillin/streptomycin mixture of 100 U/mL). Following three consecutive generations of cell culture (48 hours per generation), stable Mac-T cells were resuspended in a complete culture medium and seeded in a six-well plate with an inoculation density of 1×10^6^ cells/well. The culture medium was discarded upon reaching the logarithmic growth phase. Subsequently, the cells were challenged with a solution of Newman strain of *S. aureus* for a duration of 6 hours, with a bacteria-to-cell ratio (multiplicity of infection) of 10:1. Following the *S. aureus* challenge, the six-well plate was washed three times with sterile phosphate-buffered saline (PBS) Subsequently, 1 mL of Trizol was added to lyse the cells in the plate, and the RNA was extracted from the lysate for library preparation and sequencing. After library preparation, RNA-seq sequencing was performed using the Illumina HiSeq2500 platform at Novogene Corporation, generating Data two in our study.

The siRNAs employed in this study included *siFTO*, and a non-targeting control siRNA (*siNC*). All siRNA transfections were carried out according to the manufacturer’s instructions using the Lipofectamine 2000 transfection reagent (ThermoFisher, Waltham, MA, USA). The interfering fragments employed in this study were specifically designed and provided by Jintuosi Company (Beijing, China). The siRNA sequence for the sense strand (5’-3’) is GCGAGUUCAGA UGGGACAUTT, and the antisense strand (5’–3’) sequence is AUGUCCCAUCUGAACUCGCTT. A cell seeding of 5×10^5^ cells per well was conducted in a six-well plate. When the cell confluence reached 60% to 80%, the complete cell culture medium was removed, and the cells were subjected to two washes with sterile PBS buffer. Subsequently, the fresh complete cell culture medium (DMEM supplemented with 10% fetal bovine serum) was added, supplemented with 100 pmol of siRNA and 5 μL of transfection reagent (lipofectamine 2000) per well. The cells were then incubated at 37°C in a cell culture incubator for a duration of 36 hours. After the incubation period, cells were lysed using TRIzol for RNA extraction. The extracted RNA was then used for library preparation and sequenced on the Illumina HiSeq2500 platform provided by Novogene Company, which constituted dataset six.

### Quantitative real-time polymerase chain reaction

The quantitative real-time polymerase chain reaction (qRT-PCR) procedure for target genes was performed as follows. Total RNA was extracted using the Trizol reagent. The concentration and quality of RNA was assessed using a Nanodrop 2000 spectrophotometer. After confirming the primer specificity through conventional PCR, qRT-PCR was performed with two technical replicates for each sample. The average Ct value of the technical replicates for each sample was used for subsequent analysis. The 2^−ΔΔCt^ method [26] was employed to calculate the relative expression levels of the target genes among the samples.

### Western blot

Cells were collected from a single well of a six-well plate and lysed with a mixture of radio immunoprecipitation assay (Beyotime, Shanghai, China; P0013B) buffer and phenylmethylsulfonyl fluoride (Solarbio, Beijing, China; P0100) (100:1, 200 μL). After centrifugation at 12,000 g, the protein concentration in the supernatants was determined using the Beyotime Enhanced BCA Protein Assay Kit (P0010). The samples were appropriately diluted and mixed with 5× sodium dodecyl sulfate, followed by boiling at 95°C for 10 minutes to denature the proteins. Solarbio Precast gels (PG01215-S) were employed for protein separation based on molecular weight, and the proteins were transferred onto a 0.45 μm polyvinylidene fluoride membrane using a wet transfer apparatus. The membrane was then blocked with 5% non-fat dry milk in 1× TBST (Tris 10 mM, NaCl 150 mM, 0.05% (v/v) Tween-20, pH 7.5) at room temperature for 2 hours. Subsequently, the membrane was incubated overnight at 4°C with primary antibodies, including Anti-GAPDH antibody (1:3,000; Solarbio, China; K200057M) and Anti-FTO antibody (1:1,000; Abcam, Cambridge, UK; EPR24440-12). After washing five times with 1× TBST for 5 minutes each, the membrane was incubated with a goat anti-rabbit immunoglobulin G (IgG) secondary antibody (1:3,000; Beyotime, China; A0208) for 2 hours at room temperature. The membrane was washed five times with TBST for 5 minutes each. Finally, the membrane was visualized using the BeyoECL Plus chemiluminescent substrate (P0018S).

### Statistical analysis

Complementary statistical evaluations were conducted using GraphPad Prism version 9.3.1. RMRGs exhibiting consistent expression across over five challenge groups were further selected for their trends. By calculating the average value for each group, a singular paired t-test was carried out, wherein a p-value less than 0.05 was taken as indicative of statistical significance.

## RESULTS

### Immune-related QTLs neighbor the mammalian RMRGs

**Figure 2:**
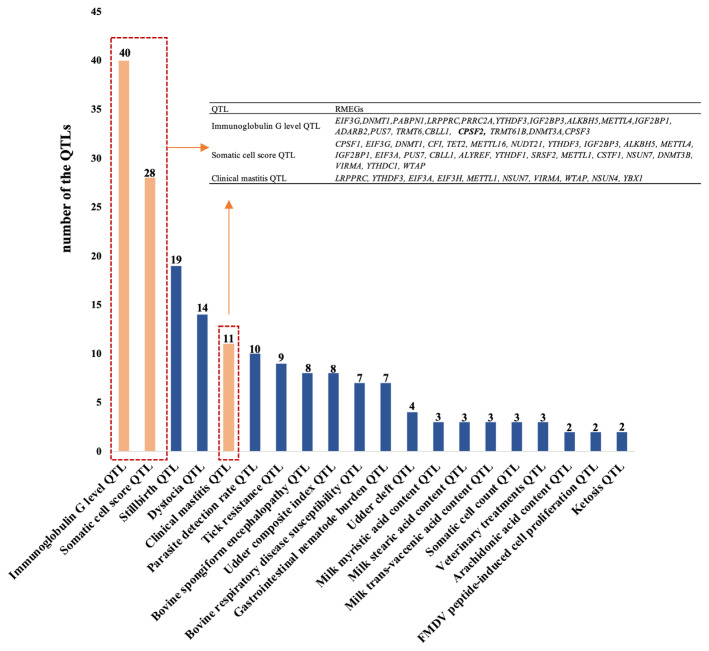
The top 20 immunity-associated quantitative trait loci (QTLs) identified by RNA modification-related genes.

To explore the roles of RMRGs in cattle complex traits, 80 genes selected in this study were evaluated by mapping QTLs in public databases (Supplementary Table S1). As shown in Figure 2, a total of 186 immune-related QTLs were identified within 1 kb upstream and downstream of the RNA modification-related enzyme genes, including QTLs associated with IgG level, somatic cell score (SCS), and clinical mastitis. The QTLs of SCC neighbor 22 RMRGs, including *CPSF1*, *TET3*, *EIF3G*, *CFI*, *TET2*, *METTL16*, *NUDT21*, *LRPPRC*, *YTHDF3*, *ALKBH5*, *METTL4*, *IGF2BP1*, *EIF3A*, *PUS7*, *CBLL1*, *SRSF2*, *CSTF1*, *NSUN7*, *DNMT3B*, *VIRMA*, *DNMT3A*, and *YTHDC1*. The RMRGs that identified the QTLs for clinical mastitis included *LRPPRC*, *YTHDF3*, *EIF3A*, *EIF3H*, *METTL1*, *NSUN7*, *VIRMA*, *WTAP*, *NSUN4*, and *YBX1*. Furthermore, a large number of immune-related QTLs also neighbor RMRGs in pig and sheep (Supplementary Figure S1). Collectively, these RMRGs may play an important role in animal immunity traits.

Mapping analysis of TWAS database was further performed on the bovine RMRGs. As shown in Table 2, the gene expression of RNA modifying-related enzymes is associated with immune system activation and plays a role in regulating responses to disease. For example, *HAKAI* was associated with mastitis, *METTL3* with metritis, and *HNRNPA2B1* and *NOP2* with somatic cell score. Those results further showed the functions of RMRGs in regulating the bovine immune response.

### Expression of RMRGs can well characterize bovine *S. aureus* mastitis

**Figure 3:**
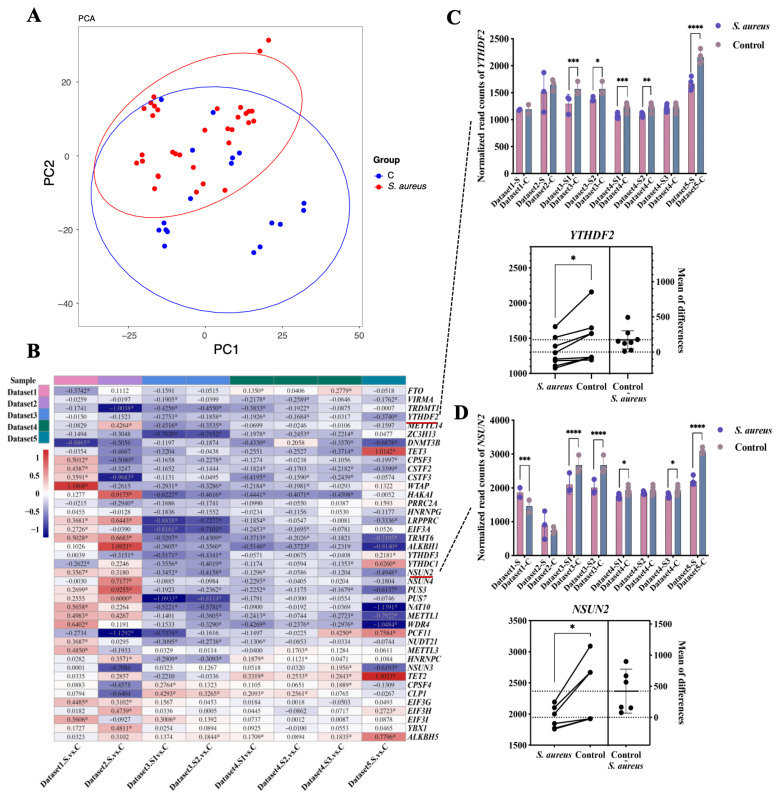
Principal component analysis (PCA) and Log_2_fold change (FC) for gene expression of RNA modification-related genes in control and *Staphylococcus aureus* challenged groups. (A) presents the PCA results of control and *S. aureus* challenged groups from all datasets. (B) presents Log_2_FC between the *S. aureus* challenged group and control group in each data. * Means the p-value of less than 0.05. (C)-(D) Presents the normalized expression levels of the *NSUN2* and *YTHDF2* genes across eight distinct groups. * p<0.05, ** p<0.01, *** p<0.001, **** p<0.0001. Paired t-test representing the significantly consistent trend group of gene changes.

To investigate the functions of RMRGs in the immune response of bovine *S. aureus* mastitis, the downloaded RNA-seq datasets related to bovine *S. aureus* mastitis from public databases were subjected to unified standardized data processing. Genes associated with RNA modification enzymes were then selected for subsequent analysis. PCA of all the data shows that the expression of RMRGs could well distinguish the control and *S. aureus* challenged groups (Figure 3A; Supplementary Figure S2). In dataset one, the *S. aureus* challenge caused physiological and morphological changes, such as increased body temperature, SCC at the individual-level. These individual-level changes are consistent with the clinical feature of mastitis in dairy cows, and the differential expression of RMRGs is a significant response to the *S. aureus* challenge in bovine mastitis.

There were eight strains of *S. aureus* involved in these five datasets. For RMRGs, only that with the same change direction of differential expression in more than five strains challenged groups were considered to be important *S. aureus* mastitis-related genes. In total, 41 important genes were selected from the 80 RMRGs (Table 3; Figure 3B). Large differences were found in the expression patterns between individual-level and cell-level, while the change trend of RNA-changed gene expression at the cellular level was almost consistent across the different datasets. Among 41 selected RMRGs, the expression levels of 29 genes were decreased after the *S. aureus* challenged treatment. In this study, some significant differences of expression level between the *S. aureus* challenged and control groups were observed by employing paired t-test, such as *YTHDF2* and *NSUN2* presented in Figure 3C–3D. These findings indicate that RMRGs may collectively contribute to the regulation of inflammatory responses in bovine *S. aureus* mastitis.

### Significant correlation of expression among RMRGs

Based on dataset four, WGCNA were constructed to further investigate the interactions of RMRGs. The top 10,000 genes with highly variable expression were clustered into 15 modules, including 14 co-expression modules and one module consisting of other genes.

**Figure 4:**
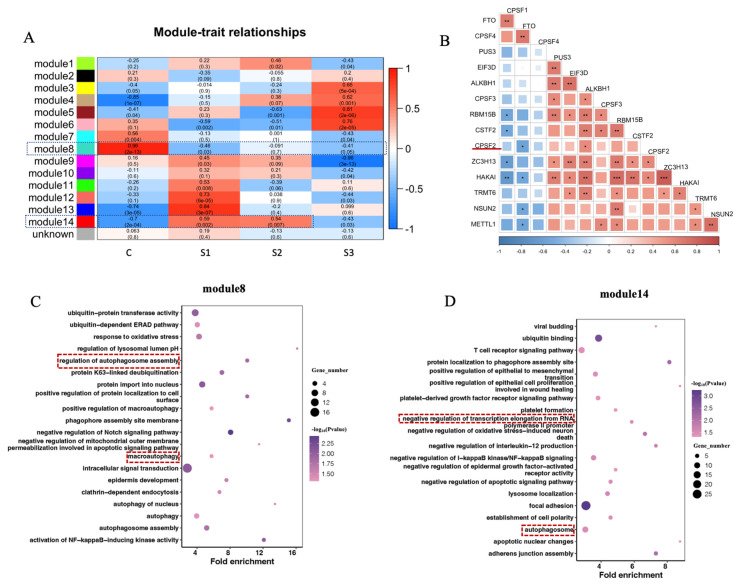
RMRGs exhibit interactions and participate in immune regulation. (A) Weighted correlation network analysis for different *Staphylococcus aureus* challenged groups. (B) Correlation of gene expression among RMRGs within Module 8. (C)–(D) Gene ontology analysis of the genes for modules 8 and 14. RMRGs, RNA modification-related genes. * p<0.05, ** p<0.01, and *** p<0.001, respectively.

The relationship between the expression of the module gene and the treatment groups was analyzed. In module 8, the changes in the challenged S1 group were more pronounced than in the S2 and S3 groups, compared to the control group (Figure 4A). In module 14, the control group had the highest positive correlation (p<0.05), while negative correlations were found with the challenged groups S1, S2, and S3, respectively. Thus, modules 8 and 14 were selected for further investigation. Within modules 8 and 14, there are many types of enzyme genes correlated with RNA modifications. There was the largest number of RNA modified gene enriched in module 8 (Table 4), including m^6^A modified writer (*ZC3HB*, *RBM15B*, and *HAKAI*), eraser (*FTO*), and reader (*EIF3D*); m^5^C modified writer (*NSUN2*), and reader (*ALKBH1*); m^1^A modified writer (*TRMT6*), the reader (*ALKBH1*), the eraser (*FTO*); writers of A-to-I (*CPSF1*, *CPSF2*, *CPSF3*, *CPSF4*, and *CSTF2*); writers of ψ (*PUS3*); writer of m^7^G (*METTL1*). Due to similar expression patterns, enzyme genes correlated with RNA modifications from various types were clustered into a same module, which suggested potential interactions among RMRGs.

To further investigate the relationships among RMRGs, expression correlations of selected genes from module 8 were calculated based on dataset four (Figure 4B). Significant positive correlations were observed in 39 gene pairs, while significant negative correlations were observed in 8 gene pairs. Interactions were found among genes from a same modification, such as a significant negative correlation between the m^6^A writer *HAKAI* and the m^6^A eraser *FTO*. Interactions between different RNA modifications were also identified, including a significant negative correlation between the m^6^A eraser *FTO* and the m^5^C writer *NSUN2*, as well as a significant negative correlation between *FTO* and the APA writer *CPSF2*. Similar results were found for other modules (Supplementary Figure S3). These findings suggest potentially synergistic or antagonistic effects of RNA modifications in bovine *S. aureus* mastitis.

The GO enrichment analysis demonstrated that genes within modules 8 and 14 are closely associated with the *S. aureus* challenge, and some significant (p<0.05) terms associated with immunity and inflammation were found. For example, GO terms macroautophagy, apoptotic process, and mRNA methylation were enriched in module 8 (Figure 4C), and terms RNA splicing, negative regulation of epithelial cell migration, apoptosis process, and cell adhesion molecule binding were enriched in module 14 (Figure 4D). In this study, different RNA epigenetic modifications can be clustered in the same co-expression module and are associated with immune responses.

### Interactions between RNA modifications are partly explained by the existence of m^6^A modification within RMRGs

In this study, a significant correlation between RMRGs, however, the mechanism behind this has to be investigated further. The genes within module 8 showed the strongest positive correlation and the largest number of clustered genes in the control group based on the WGCNA enrichment analysis. Importantly, this module also exhibited the highest abundance of RMRGs among all the identified modules. m^6^A modification is extensively studied in RNA modification research, with *FTO* playing a significant role as an m^6^A demethylase in its intricate regulation. This study supports the association of *FTO* with *S. aureus* mastitis at the individual level within dataset one. Thus, we chose *FTO*, a gene in module 8 for validation. To confirm the connection of gene expression between RMRGs, an RNA interference experiment was performed for *FTO*.

**Figure 5:**
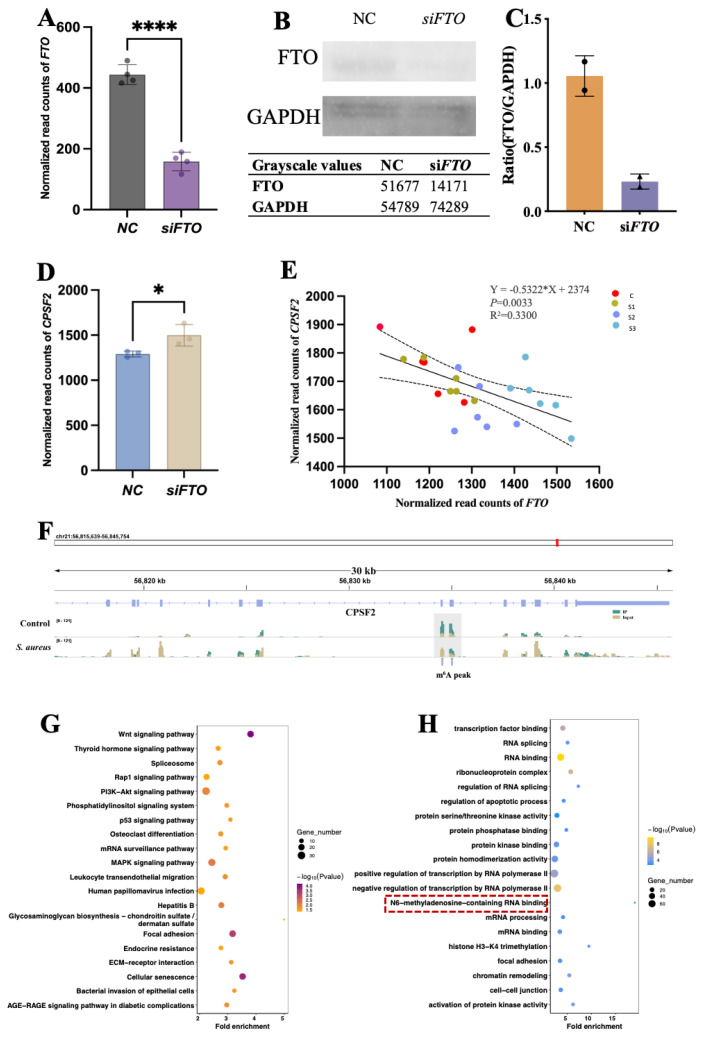
*FTO* influence the expression of RNA-modified genes containing m^6^A modification sites. (A) Interference result for *FTO*. **** p<0.001. (B)–(C) Total cell extracts were harvested from si*FTO* and NC Mac-T cells and subjected to Western blot. (D) Expression changes of *CPSF2* gene after cellular-level interference with *FTO*. * p<0.05. (E) *CPSF2* gene expression significantly correlated with *FTO*. (F) Variations in the m^6^A modification peak within the *CPSF2* gene between the control group and *Staphylococcus aureus* challenged group. (G) Kyoto encyclopedia of genes and genomes analysis of differentially expressed genes (DEGs) with m^6^A peaks post-*FTO* interference. Circle attributes indicate p-value significance and gene count. (H) Gene ontology enrichment of DEGs with m^6^A peaks post-*FTO* interference. Circle attributes indicate p-value significance and gene count. m^6^A, N6-methyladenosine.

According to the interference experiments, siRNA significantly (p<0.0001) knocked down the expression and protein level of *FTO* (Figure 5A–5C). Interference of gene *FTO* significantly increased the expression level of *CPSF2* (Figure 5D), which confirmed the negative correlation between *FTO* and *CPSF2* found in this study (Figure 4B). Furthermore, through analysis of downloaded MeRIP-seq data, we identified the presence of m^6^A modification on the *CPSF2* gene, and the m^6^A peak decreased after *S. aureus* challenged. (Figure 5F). Therefore, *FTO* likely regulates the expression of *CPSF2* by modulating its m^6^A modification. Based on our findings, the *FTO* gene affects the expression of RMRGs that harbor m^6^A modification sites, which is likely attributed to the presence of m6A modifications within the genes involved in RNA modification. The initial findings on the function of RMRGs in *S. aureus* mastitis and the role of *FTO* in this biological process have the potential to partially elucidate the molecular mechanisms responsible for S. aureus mastitis in bovine.

In integration of MeRIP-seq and RNA-seq following FTO gene interference, it found a set of 3,224 DEGs, of which 1,164 DEGs exhibited m6A modification peaks. The KEGG pathway enrichment (Figure 5G) revealed a significant enrichment of these m6A-marked DEGs in inflammation-associated pathways, such as the Wnt signaling pathway, the Measles pathway, and the Hepatitis B pathway. GO enrichment analysis for 1,164 m6A-marked DEGs (Figure 5H) revealed a strong enrichment of m6A marked genes in biological processes and molecular functions related to transcription factor binding, RNA splicing and RNA binding. The potential impact of FTO on processes related to transcriptional regulation and post-transcriptional modification was emphasized by the prominence of categories related to transcriptional regulation and post-transcriptional modification. Interestingly, the term associated with “m^6^A-methyladenosine-containing RNA binding” was also enriched. These findings show how RNA modifications can affect bovine *S. aureus* mastitis, by illustrating the potential underlying molecular mechanisms resulting from *FTO* interference.

## DISCUSSION

This study revealed the association between RMRGs and the immune system, specifically in bovine *S. aureus* mastitis. Analysis of RNA-seq data from five datasets showed consistency within challenged and control groups at individual and cellular levels, as well as heterogeneity between groups. We identified 41 key genes with consistent differential expression patterns across datasets, and correlation analysis demonstrated interactions between homologous and different RNA modifications. Furthermore, we validated the regulation of RMRGs at the cell level using MeRIP-seq data and the interference experiment, providing additional evidence for potential interactions between different RNA modifications.

For bovine *S. aureus* mastitis, we conducted an analysis of the ten well-characterized RNA modifications. The key genes identified by the current study accounted for more than 50% of the m^1^A, m^5^C, and m^6^A modified genes reported in previous studies. Therefore, the m^1^A, m^5^C, and m^6^A modifications should be given special attention in studies of bovine *S. aureus* mastitis. Previous studies have indicated that m^5^C modification is associated with the development of systemic lupus erythematosus through its influence on CD4+ T cells involved in immune responses [27]. A-to-I and m^6^A modifications have regulatory functions in human cardiovascular disease [28,29]. Furthermore, RNA modification plays a crucial role in maintaining immune cell homeostasis and function [30,31]. Notably, m^6^A modification, regulated by the m^6^A reading protein *YTHDF2*, suppresses circRNA-mediated immune responses in humans [32]. Additionally, genes modified by m^6^A are enriched in several immune signaling pathways in the bovine *Escherichia coli* mastitis [33]. In this study, the regulatory role of RNA modifications in immune complex traits were investigated by bovine QTL and TWAS databases. This study identified a strong association between RMRGs and immune complex traits in cattle, such as clinical mastitis. PCA revealed distinct clustering patterns of RMRGs between the *S. aureus* challenged group and the control group, indicating their essential role in bovine *S. aureus* mastitis. Consistent changes in the expression of RMRGs were also observed across different cellular-level RNA-seq datasets. It is necessary to further investigate the mechanisms underlying RNA modification genes in *S. aureus* bovine mastitis.

Recent studies have indicated that m^6^A modification exerts an influence on immune regulation by regulating processes, such as RNA alternative splicing and APA [34]; thereby impacting the expression and function of immune-related genes [35]. *FTO* plays a crucial role in controlling APA and subsequently determining the length of 3’ UTRs, as evidenced by the up-regulated expression of the last exon in cells lacking *FTO* [36]. CPSF2 is a member of the protein complex associated with RNA 3’ end processing and is involved in the APA process [37,38]. In the present study, interference with the *FTO* gene significantly increased the expression level of *CPSF2* containing m^6^A modification locus; and the m^6^A level in *CPSF2* was reduced after *S. aureus* challenge. Consequently, we hypothesize that *FTO*, through its m^6^A modification, may regulate the expression of *CPSF2* and be involved in the APA process in response to immune regulation. In the future, more thorough work to understand the underlying mechanisms of these interactions would be essential, aiming to gain a comprehensive understanding of how m^6^A modification collaboratively contributes to immune regulation through APA.

This study revealed potential interactions between enzymes involved in RNA modifications within the same category and across different categories. We propose a mechanism involving RNA modification genes that regulate these interactions. The importance of RNA modification interactions in immune regulation was supported by many studies in various species [10]. For example, the regulation of RNA methylation (m^1^A, m^5^C, m^6^A, and m^7^G) in brain tissue damage was explored using zebrafish, revealing specific changes in these modifications under hypoxic conditions [39]. Crosstalk mechanisms were also investigated in colon cancer treatment, and the regulatory role of different RNA modifications and their impact on the immune microenvironment were reported [40,41]. In hepatocellular carcinoma, it was shown the regulation of immune-related genes through protein-protein interactions [41,42]. Interaction studies in these species provide support for findings of this study, suggesting the need for in-depth exploration of the mechanisms governing the interplay of RNA modifications in bovine immune regulation.

Compared to the cellular level, the expression changes of RMRGs for bovine *S. aureus* mastitis showed slight differences at the individual level. These inconsistencies may be due to the different environments of cell lines and individual tissues. Based on our findings, we cautiously speculate that there are three possible causes: on the first hand, the results at the individual level are less consistent with the phenomenon at the cellular level due to the influence of only two individual cows; on the next hand, individual-level mammary tissue contains various classes of cell types and the mechanisms regulating phenotypes in individuals are complex multifactorial influences [3]; on the other hand, the sampling at the individual-level has multiple influences and is variable in time-space compared to the cellular level. Therefore, genes with strong consistency between the individual and cellular levels can be selected as stable genetic markers for disease-resistance breeding in bovines.

In conclusion, RNA modifications were found to play an important role in bovine *S. aureus* mastitis. The effect of different *S. aureus* strains on the expression of RMRGs varied after challenge with mammary epithelial cells. RMRGs can be used as potential indicators to assess the status of bovine mastitis. Focusing on the interactions between RMRGs is essential to reveal the genetic architecture of complex traits. The findings from this study are beneficial for understanding the regulatory mechanism of RMRGs on complex traits in cattle.

## Figures and Tables

**Table 1:** The grouping information for all datasets

Sample	The sample record number of sequence read archive experiment			
Dataset one	SRX1713237 C-1	SRX1713500 S-1		
	SRX1713308 C-2	SRX1713526 S-2		
Dataset two	SRX11650574 C-1	SRX11650571 S-1		
	SRX11650575 C-2	SRX11650572 S-2		
	SRX11650576 C-3	SRX11650573 S-3		
Dataset three	SRX13089400 C-1	SRX13089403 S1-1	SRX13089406 S2-1	
	SRX13089401 C-2	SRX13089404 S1-2	SRX13089407 S2-2	
	SRX13089402 C-3	SRX13089405 S1-3	SRX13089408 S2-3	
Dataset four	SRX10643265 C-1	SRX10643269 S1-1	SRX10643282 S2-1	SRX10643295 S3-1
	SRX10643266 C-2	SRX10643270 S1-2	SRX10643283 S2-2	SRX10643296 S3-2
	SRX10643277 C-3	SRX10643271 S1-3	SRX10643284 S2-3	SRX10643297 S3-3
	SRX10643288 C-4	SRX10643272 S1-4	SRX10643285 S2-4	SRX10643298 S3-4
	SRX10643299 C-5	SRX10643273 S1-5	SRX10643286 S2-5	SRX10643300 S3-5
	SRX10643308 C-6	SRX10643274 S1-6	SRX10643287 S2-6	SRX10643301 S3-6
Dataset five	SRR24210509 C-1	SRR24210505 S-1		
	SRR24210508 C-2	SRR24210504 S-2		
	SRR24210507 C-3	SRR24210503 S-3		
	SRR24210506 C-4	SRR24210502 S-4		
Dataset six	SRR24210509 C-1	SRR26403327 *siFTO*-1		
	SRR24210508 C-2	SRR26403326 *siFTO*-2		
	SRR24210507 C-3	SRR26403325 *siFTO*-3		
	SRR24210506 C-4	SRR26403324 *siFTO*-4		
Dataset seven	SRR13005820 Input-1	SRR13005811 IP-1		
	SRR13005821 Input-2	SRR13005812 IP-2		
	SRR13005822 Input-3	SRR13005813 IP-3		

Note: The underlined datasets were generated by this study. For six RNA-seq datasets, dataset one is at the individual level, whereas dataset two to six are at the cellular level. The MeRIP-seq dataset is dataset seven. “C” represents the control, “S” represents the challenge of *S. aureus*, and “*siFTO*” represents the group with *FTO* gene interference. “Input” represents the total RNA before immunoprecipitation, serving as a control. “IP” represents “Immunoprecipitation,” which is the fraction of RNA enriched for m^6^A modifications.

**Table 2:** Trait prediction of RMRGs in TWAS data

RNA modification	Gene	Tissue	Trait	Zscore^1)^	p-value	Pred_Perf_Pval^2)^
m^6^A	*METTL3*	Liver	Metritis	−2.3909	0.0168	0.0028
	*VIRMA*	Intramuscular_fat	Foot angle	−2.4427	0.0146	0.0133
	*RBM15B*	Blood	Rear teat placement	−2.6011	0.0093	0.0070
	*HAKAI*	Blood	Mastitis	2.4212	0.0155	0.0060
	*ALKBH5*	Jejunum	Heifer conception rate	−2.4593	0.0139	0.0137
	*YTHDF1*	Intramuscular_fat	Daughter pregnacy rate	4.1217	<0.0001	0.0038
	*IGF2BP3*	Mammary	Displaced abomasum	−2.5360	0.0112	0.0370
	*PRRC2A*	Liver	Age at first calving	−3.6954	0.0002	<0.0001
	*HNRNPA2B1*	Lymph_node	Somatic cell score	−3.2516	0.0011	0.0175
	*HUR*	Blood	Feet and legs composite	2.6296	0.0085	<0.0001
	*EIF3A*	Intramuscular_fat	Front teat placement	3.4039	0.0007	<0.0001
	*EIF3B*	Liver	Rump width	−2.3750	0.0175	0.0009
	*EIF3D*	Hypothalamus	Protein percentage	2.4948	0.0126	0.0020
	*EIF3G*	Adipose	Fore udder attachment	−2.4327	0.0150	0.0207
	*EIF3H*	Intramuscular_fat	Body depth	2.8686	0.0041	0.0119
A-To-I	*ADAR*	Lung	Protein percentage	−2.7649	0.0057	0.0018
	*ADARB2*	Lymph_node	Protein percentage in milk	−2.6487	0.0081	<0.0001
m^1^A	*TRMT61A*	Blood	Dairy form	−1.9728	0.0485	0.0045
	*YTHDF1*	Intramuscular_fat	Daughter pregnacy rate	4.1217	<0.0001	0.0038
m^5^C	*NSUN1*	Blood	Somatic cell score	2.7819	0.0054	<0.0001
	*NSUN3*	Liver	Hypocalcemia	−2.7430	0.0061	0.0017
	*NSUN6*	Adipose	Ketosis	1.9764	0.0481	0.0015
	*TET1*	Macrophage	Body depth	−1.9606	0.0499	0.0002
	*TET2*	Muscle	Daughter still birth	−2.3387	0.0193	0.0062
	*ALYREF*	Blood	Stature	2.7530	0.0059	<0.0001
ψ	*PUS3*	Blood	Sire still birth	−2.1355	0.0327	0.0016
hm^5^C	*TET2*	Muscle	Daughter still birth	−2.3387	0.0193	0.0062
m^7^G	*METTL1*	Liver	Net merit	3.5597	0.0004	0.0021
APA	*CPSF1*	Macrophage	Fat percentage in milk	−16.3369	<0.0001	<0.0001
	*CPSF3*	Mammary	Stature	2.4495	0.0143	0.0319
	*CPSF4*	Macrophage	Rump width	3.1984	0.0014	0.0011
	*CSTF3*	Lymph_node	Rump angle	−2.4693	0.0135	0.0037

RMRGs, RNA modification-related genes; TWAS, transcriptome-wide association studies; m^6^A, N6-methyladenosine; m^6^Am, N6, 2’-O-dimethyl adenosine; m^1^A, N1-methyladenosine; m^5^C, 5-methylcytosine; hm^5^C, 5-hydroxymethylcytosine; ac^4^C, N4-acetyl cytidine; APA, alternative polyadenylation; Ψ, pseudouridine; m^7^G, N7-methylguanosine.

1) Zscore: S-PrediXcan’s association result for the gene, typically HUGO for a gene.

2) Pred_Perf_Pval: p-value of tissue model’s correlation to gene’s measured transcriptome (prediction performance).

**Table 3:** RMRGs chosen in this study^1)^

RNA modification	*S. aureus* challenged	Writer	Eraser	Reader
m^6^A (18/34)	Up-regulated		*ALKBH5*	*HNRNPC, FMR1, EIF3G, EIF3H, EIF3I*
	Down-regulated	*METTL14, WTAP, VIRMA, ZC3H13, HAKAI*		*YTHDC1, YTHDC2, YTHDF2, YTHDF3, HNRNPG, LRPPRC, EIF3A*
	Non-significant	*METTL3, METTL16, RBM15, RBM15B*	*FTO*	*YTHDF1, IGF2BP1, IGF2BP2, IGF2BP3, PRRC2A, HNRNPA2B1, SRSF2, HUR, EIF3B, EIF3C, EIF3D*
m^5^C (9/17)	Up-regulated	*NSUN3*	*TET2*	*YBX1*
	Down-regulated	*NSUN2, NSUN6, DNMT2, DNMT3B*	*TET3*	*YTHDF2*
	Non-significant	*NSUN1, NSUN4, NSUN5, NSUN7, DNMT1, DNMT3A*	*TET1*	*ALYREF*
m^1^A (6/13)	Down-regulated	*TRMT6*	*ALKBH1*	*YTHDF2, YTHDF3, YTHDC1, YTHDC2*
	Non-significant	*TRMT61A, TRMT61B, TRMT10C, NML*	*ALKBH3, FTO*	*YTHDF1*
m^6^Am (1/3)	Down-regulated	*METTL4*		
	Non-significant	*PCIF1*	*FTO*	
Adenosine-to-inosine editing (A-to-I)	Non-significant	*ADAR, ADARB1, ADARB2*		
Pseudouridine (ψ) (2/7)	Down-regulated	*PUS3, PUS7*		
	Non-significant	*PUS1, PUS2, PUS4, PUS6, PUS9*		
ac^4^C (1/1)	Down-regulated	*NAT10*		
m7G (2/2)	Down-regulated	*METTL1, WDR4*		
APA (Alternative polyadenylation)(8/12)	Up-regulated	*CPSF1, CPSF4, CLP1*		
	Down-regulated	*CPSF3, CSTF2, CSTF3, PCF11, NUDT21*		
	Non-significant	*CPSF2, CSTF1, CFI, PABPN1*		
hm5C (1/1)	Up-regulated	*TET2*		

RMRGs, RNA modification-related genes.

1) The percentage of RMRGs showing consistent modifications, as classified by our analysis, is presented in brackets alongside the respective modification genes.

**Table 4:** The distribution of different RMRGs in different modules

Item	In-module gene counts	RMRGs count	RMRGs
module8	1,887	15	*ZC3H13, RBM15B, HAKAI, FTO, EIF3D, TRMT6, ALKBH1, NSUN2, PUS3, METTL1, CPSF1, CPSF2, CPSF3, CPSF4, CSTF2*
module13	1,328	12	*HNRNPA2B1, HNRNPC, HUR, LRPPRC, EIF3A, TRMT10C, ALKBH3, YBX1, PUS1, NAT10, WDR4, CSTF3*
module11	760	9	*ALKBH5, YTHDC1, EIF3H, EIF3I, PCIF1, NSUN6, DNMT3B, ALYREF, PUS7*
module14	685	7	*METTL16, WTAP, VIRMA, YTHDF2, NSUN1, CLP1, NUDT21*
module5	1,311	5	*YTHDF1, SRSF2, NSUN3, DNMT1, DNMT3A*
module2	636	5	*YTHDC2, EIF3C, EIF3G, NML, TET3*
module3	942	3	*RBM15, IGF2BP2, TET2*
module6	599	3	*PRRC2A, HNRNPG, ADARB1*
unknown	295	3	*METTL14, YTHDF3, CSTF1*
module12	114	3	*NSUN4, DNMT2, TRDMT1*
module10	415	2	*FMR1, ADAR*
module1	259	2	*EIF3B, NSUN5*
module4	181	2	*METTL3, PABPN1*
module9	490	1	*PCF11*
module7	98	1	*TRMT61A*

RMRGs, RNA modification-related genes.

## Data Availability

All raw and processed sequencing data generated in this study have been submitted to the NCBI Sequence Read Archive (SRA; https://www.ncbi.nlm.nih.gov/sra) under accession number PRJNA957225 ( https://www.ncbi.nlm.nih.gov/bioproject/PRJNA957225).

## SUPPLEMENTARY MATERIAL

**Supplementary Figure S1**. The immunity-associated QTLs identified by RMRGs in pig and sheep

**Supplementary Figure S2**. (A) presents the PCA results of control and S. aureus challenged groups. (B)–(F) presents the PCA results of dataset one to five, respectively.

**Supplementary Figure S3**. Correlation of gene expression among RMRGs within Module 14.

**Supplementary Table S1**. The RMRGs selected for this research

## References

[1] Becker K, Schaumburg F, Kearns A (2019). Implications of identifying the recently defined members of the Staphylococcus aureus complex S. argenteus and S. schweitzeri: a position paper of members of the ESCMID Study Group for Staphylococci and Staphylococcal Diseases (ESGS). Clin Microbiol Infect.

[2] Chan CTY, Dyavaiah M, DeMott MS, Taghizadeh K, Dedon PC, Begley TJ (2010). A quantitative systems approach reveals dynamic control of tRNA modifications during cellular stress. PLoS Genet.

[3] Mi S, Tang Y, Dari G (2021). Transcriptome sequencing analysis for the identification of stable lncRNAs associated with bovine Staphylococcus aureus mastitis. J Anim Sci Biotechnol.

[4] Chen Y, Yang J, Huang Z (2022). Exosomal lnc-AFTR as a novel translation regulator of FAS ameliorates Staphylococcus aureus-induced mastitis. Biofactors.

[5] Huynh HT, Robitaille G, Turner JD (1991). Establishment of bovine mammary epithelial cells (MAC-T): an in vitro model for bovine lactation. Exp Cell Res.

[6] Ogunnaike M, Wang H, Zempleni J (2021). Bovine mammary alveolar MAC-T cells afford a tool for studies of bovine milk exosomes in drug delivery. Int J Pharm.

[7] Günther J, Koy M, Berthold A, Schuberth HJ, Seyfert HM (2016). Comparison of the pathogen species-specific immune response in udder derived cell types and their models. Vet Res.

[8] Gilbert WV, Bell TA, Schaening C (2016). Messenger RNA modifications: form, distribution, and function. Science.

[9] Roundtree IA, Evans ME, Pan T, He C (2017). Dynamic RNA modifications in gene expression regulation. Cell.

[10] Zhang M, Song J, Yuan W, Zhang W, Sun Z (2021). Roles of RNA methylation on tumor immunity and clinical implications. Front Immunol.

[11] Song P, Tayier S, Cai Z, Jia G (2021). RNA methylation in mammalian development and cancer. Cell Biol Toxicol.

[12] Chen H, Gu L, Orellana EA (2020). METTL4 is an snRNA m(6)Am methyltransferase that regulates RNA splicing. Cell Res.

[13] Elkon R, Ugalde AP, Agami R (2013). Alternative cleavage and polyadenylation: extent, regulation and function. Nat Rev Genet.

[14] He C, Bozler J, Janssen KA (2021). TET2 chemically modifies tRNAs and regulates tRNA fragment levels. Nat Struct Mol Biol.

[15] He PC, He C (2019). mRNA acetylation: a new addition to the epitranscriptome. Cell Res.

[16] Nishikura K (2016). A-to-I editing of coding and non-coding RNAs by ADARs. Nat Rev Mol Cell Biol.

[17] Nombela P, Miguel-López B, Blanco S (2021). The role of m(6)A, m(5)C and Ψ RNA modifications in cancer: novel therapeutic opportunities. Mol Cancer.

[18] Safra M, Sas-Chen A, Nir R (2017). The m1A landscape on cytosolic and mitochondrial mRNA at single-base resolution. Nature.

[19] Selmi T, Hussain S, Dietmann S (2021). Sequence- and structure-specific cytosine-5 mRNA methylation by NSUN6. Nucleic Acids Res.

[20] Yang X, Yang Y, Sun BF (2017). 5-methylcytosine promotes mRNA export - NSUN2 as the methyltransferase and ALYREF as an m(5)C reader. Cell Res.

[21] Zhang LS, Liu C, Ma H (2019). Transcriptome-wide mapping of internal N(7)-Methylguanosine methylome in mammalian mRNA. Mol Cell.

[22] Li W, Zhang X, Lu X (2017). 5-Hydroxymethylcytosine signatures in circulating cell-free DNA as diagnostic biomarkers for human cancers. Cell Res.

[23] Patel RK, Jain M (2012). NGS QC Toolkit: a toolkit for quality control of next generation sequencing data. PLoS One.

[24] Kim D, Langmead B, Salzberg SL (2015). HISAT: a fast spliced aligner with low memory requirements. Nat Methods.

[25] Leek JT, Johnson WE, Parker HS, Jaffe AE, Storey JD (2012). The sva package for removing batch effects and other unwanted variation in high-throughput experiments. Bioinformatics.

[26] Livak KJ, Schmittgen TD (2001). Analysis of relative gene expression data using real-time quantitative PCR and the 2(-Delta Delta C(T)) method. Methods.

[27] Zhernakova A, van Diemen CC, Wijmenga C (2009). Detecting shared pathogenesis from the shared genetics of immune-related diseases. Nat Rev Genet.

[28] Delatte B, Wang F, Ngoc LV (2016). Transcriptome-wide distribution and function of RNA hydroxymethylcytosine. Science.

[29] Gatsiou A, Stellos K (2023). RNA modifications in cardiovascular health and disease. Nat Rev Cardiol.

[30] Esteve-Puig R, Climent F, Piñeyro D (2021). Epigenetic loss of m1A RNA demethylase ALKBH3 in Hodgkin lymphoma targets collagen, conferring poor clinical outcome. Blood.

[31] Yang H, Wang Y, Xiang Y (2022). FMRP promotes transcription-coupled homologous recombination via facilitating TET1-mediated m5C RNA modification demethylation. Proc Natl Acad Sci USA.

[32] Luo J, Wang F, Sun F (2021). Targeted inhibition of FTO demethylase protects mice against LPS-induced septic shock by suppressing NLRP3 inflammasome. Front Immunol.

[33] Li T, Lin C, Zhu Y (2021). Transcriptome profiling of m(6)A mRNA modification in bovine mammary epithelial cells treated with Escherichia coli. Int J Mol Sci.

[34] Ke S, Pandya-Jones A, Saito Y (2017). m(6)A mRNA modifications are deposited in nascent pre-mRNA and are not required for splicing but do specify cytoplasmic turnover. Genes Dev.

[35] Bai Q, Shi M, Sun X (2022). Comprehensive analysis of the m6A-related molecular patterns and diagnostic biomarkers in osteoporosis. Front Endocrinol (Lausanne).

[36] Ke S, Alemu EA, Mertens C (2015). A majority of m6A residues are in the last exons, allowing the potential for 3’ UTR regulation. Genes Dev.

[37] Hinske LC, Galante PA, Limbeck E (2015). Alternative polyadenylation allows differential negative feedback of human miRNA miR-579 on its host gene ZFR. PLoS One.

[38] Nilubol N, Boufraqech M, Zhang L, Kebebew E (2014). Loss of CPSF2 expression is associated with increased thyroid cancer cellular invasion and cancer stem cell population, and more aggressive disease. J Clin Endocrinol Metab.

[39] Li W, Li X, Ma X, Xiao W, Zhang J (2022). Mapping the m1A, m5C, m6A and m7G methylation atlas in zebrafish brain under hypoxic conditions by MeRIP-seq. BMC Genomics.

[40] Chen H, Yao J, Bao R (2021). Cross-talk of four types of RNA modification writers defines tumor microenvironment and pharmacogenomic landscape in colorectal cancer. Mol Cancer.

[41] Li D, Li K, Zhang W (2022). The m6A/m5C/m1A regulated gene signature predicts the prognosis and correlates with the immune status of hepatocellular carcinoma. Front Immunol.

[42] Li Q, Li X, Tang H (2017). NSUN2-mediated m5C methylation and METTL3/METTL14-mediated m6A methylation cooperatively enhance p21 translation. J Cell Biochem.

