# Supplementary figures and images for: The role of RNA epigenetic modification-related genes in the immune response of cattle to mastitis induced by Staphylococcus aureus
Source: Anim Biosci. 2024 Jan 20;37(7):1141–55. doi: 10.5713/ab.23.0323 (PMC11222847; doi:10.5713/ab.23.0323)

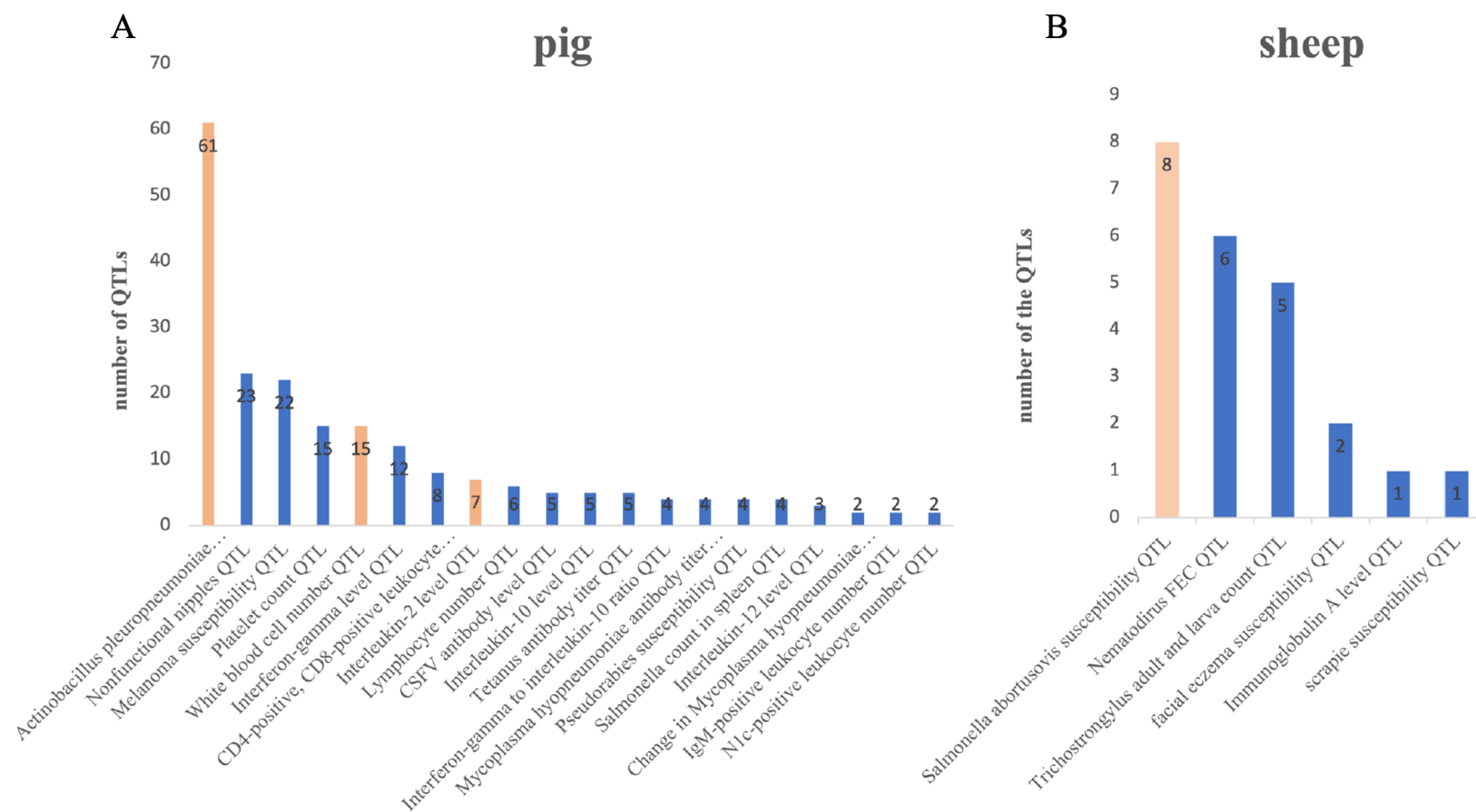

**Supplementary Figure S1** The immunity-associated QTLs identified by RMRGs in pig and sheep

Supplement: Supplementary file 1 [file ab-23-0323-Supplementary-Fig-1.pdf]
