# Supplementary material for: The role of RNA epigenetic modification-related genes in the immune response of cattle to mastitis induced by Staphylococcus aureus
Source: Anim Biosci. 2024 Jan 20;37(7):1141–55. doi: 10.5713/ab.23.0323 (PMC11222847; doi:10.5713/ab.23.0323)
Supplement: Supplementary file 2 [file ab-23-0323-Supplementary-Fig-2.pdf]

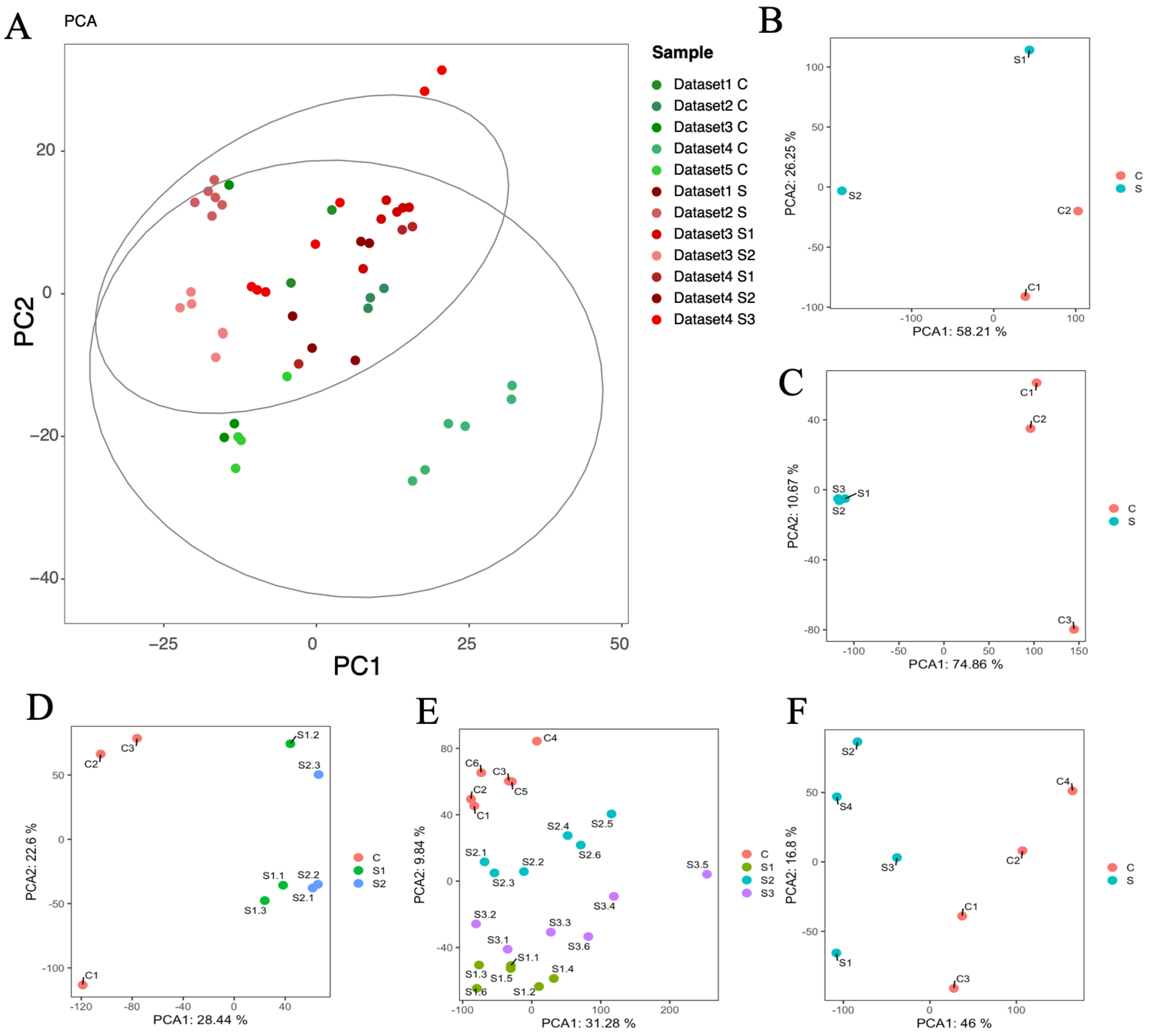

Supplementary Figure S2 A presents the PCA results of control and *S. aureus* challenged groups. **B-F** presents the PCA results of dataset one to five, respectively.
