# Supplementary material for: The role of RNA epigenetic modification-related genes in the immune response of cattle to mastitis induced by Staphylococcus aureus
Source: Anim Biosci. 2024 Jan 20;37(7):1141–55. doi: 10.5713/ab.23.0323 (PMC11222847; doi:10.5713/ab.23.0323)
Supplement: Supplementary file 4 [file ab-23-0323-Supplementary-Table-1.pdf]

Supplementary Table S1 The RMRGs selected for this research

RNA modification	writer	eraser	reader
**m^6A**	*METTL3, METTL14, METTL16, WTAP, VIRMA, ZC3H13, RBM15, RBM15B, HAKAI*	*FTO, ALKBH5*	*YTHDC1, YTHDC2, YTHDF1, YTHDF2, YTHDF3, IGF2BP1, IGF2BP2, IGF2BP3, PRRC2A, HNRNPA2B1, HNRNPC, HNRNPG, FMR1, SRSF2, HUR, LRPPRC, EIF3A, EIF3B, EIF3C, EIF3D, EIF3G, EIF3H, EIF3I*
**m^5C**	*NSUN1, NSUN2, NSUN3, NSUN4, NSUN5, NSUN6, NSUN7, DNMT1, DNMT2, DNMT3A, DNMT3B*	*TET1, TET2, TET3*	*ALYREF, YBX1, YTHDF2*
**m^1A**	*TRMT6, TRMT61A, TRMT61B, TRMT10C, NML*	*ALKBH1, ALKBH3, FTO*	*YTHDF1, YTHDF2, YTHDF3, YTHDC1, YTHDC2*
**m^6 Am**	*PCIF1, METTL4*	*FTO*	
**Adenosine-to-inosine editing (A-to-I)**	*ADAR, ADARB1, ADARB2*		
**Pseudouridine (ψ)**	*PUS1, PUS2, PUS3, PUS4, PUS6, PUS7, PUS9*		
**ac^4C**	*NAT10*		
**m^7G**	*METTL1, WDR4*		
**APA (Alternative polyadenylation)**	*CPSF1, CPSF2, CPSF3, CPSF4, CSTF1, CSTF2, CSTF3, CFI PCF11, CLP1, NUDT21, PABPN1*		
**hm^5C**	*TET2*		

Note: The RMRGs categorized by their roles in writers, erasers, and readers specific RNA modifications.
